# Strategies for the Biodegradation of Polyfluorinated Compounds

**DOI:** 10.3390/microorganisms10081664

**Published:** 2022-08-17

**Authors:** Lawrence P. Wackett

**Affiliations:** Department of Biochemistry, Molecular Biology and Biophysics and BioTechnology Institute, University of Minnesota, Minneapolis, MN 55455, USA; wacke003@umn.edu

**Keywords:** polyfluorinated, PFAS, biodegradation, bacteria, enzyme, metabolism, metabolic activation, weak bonds, defluorination

## Abstract

Many cite the strength of C–F bonds for the poor microbial biodegradability of polyfluorinated organic compounds (PFCs). However, commercial PFCs almost invariably contain more functionality than fluorine. The additional functionality provides a weak entry point for reactions that activate C–F bonds and lead to their eventual cleavage. This metabolic activation strategy is common in microbial biodegradation pathways and is observed with aromatic hydrocarbons, chlorinated compounds, phosphonates and many other compounds. Initial metabolic activation precedes critical bond breakage and assimilation of nutrients. A similar strategy with commercial PFCs proceeds via initial attack at the non-fluorinated functionalities: sulfonates, carboxylates, chlorines, phenyl rings, or phosphonates. Metabolic transformation of these non-fluorinated groups can activate the C–F bonds, allowing more facile cleavage than a direct attack on the C–F bonds. Given that virtually all compounds denoted as “PFAS” are not perfluorinated and are not alkanes, it is posited here that considering their individual chemical classes is more useful for both chemical and microbiological considerations of their fate.

## 1. Introduction

In 2022, a comprehensive search of all public sources identified more than nineteen million fluorinated chemicals, almost all of which have been created by humans within the last century [1]. Commercial polyfluorinated organic compounds (PFCs) are used in more than two hundred different applications [2]. Between 1998 and 2020, more than half of all International Organization for Standardization (ISO) approved agrichemicals were fluorinated [3]. More than 20% of new pharmaceuticals are fluorinated, mostly aromatic ring compounds [4,5]. While regulatory scrutiny is increasing, the vast majority of these fluorinated compounds remain unregulated and a knowledge of their biodegradation, and hence longevity in the environment, is widely lacking.

In light of the general lack of information on PFC biodegradation, this review draws on broad concepts of biodegradation to posit how PFCs might be transformed by natural and engineered microbes. As such, the review combines relevant literature on how microbes activate difficult-to-cleave bonds with insights from the field of organic chemistry regarding carbon–fluorine bond cleavage mechanisms. Examples with relevant references will be provided throughout the review. The present compilation differs from previous PFC biodegradation reviews that focus on a limited set of the >19 million fluorinated compounds known. For example, previous reviews have covered fluoroalkyl acids and fluorotelomers, such as those used in firefighting foams [6,7,8,9,10]. However, there are multiple classes of PFCs for which documented biodegradative metabolism is lacking. This contribution focuses on potential mechanisms by which these PFCs may be transformed by microbes based on general principles of biodegradation and organofluorine chemistry.

The vast majority, more than eighteen million, of anthropogenic fluorine compounds are aromatic [1]. While many of the commercial agrichemicals consist of multiply fluorinated aromatic ring compounds, most biodegradation studies with fluoroaromatics were with monofluorinated compounds [11,12,13]. Much remains to be learned about the microbial mechanisms by which polyfluorinated aromatic, olefinic and alkyl compounds are biodegraded. In many of the metabolic pathways for PFCs that exist or may yet evolve, this author suggests that direct C–F bond cleavage is unlikely to occur. Instead, metabolic activation of adjacent atoms can activate the C–F bond to make biodegradation feasible. This concept is documented in literature from the chemical sciences on C–F bond activation, and in literature on the activation and biodegradation of other very strong chemical bonds, such as the C–P bond of alkylphosphonic acids [14]. This author is unaware of other review articles on PFCs having this focus.

Some of the steps for the metabolic activation of PFCs may take time to evolve given that no natural product PFCs are known to humans, and so may not have entered the biosphere until very recently. This contrasts with most functional groups found in commercial chemicals that also occur in natural products that have likely existed for eons [15]. Fluorinated natural products are rare (only several dozen have been identified) and they are all monofluorinated compounds [16,17]. There are no polyfluorinated natural products known at this time. This contrasts with the synthetic and commercial production of fluorinated compounds, many of which are PFCs [1]. At one time, polychlorinated compounds were considered foreign to the biosphere, but over the past several decades, more than five thousand chlorinated natural products have been identified, many of which are polychlorinated [18]. Industry produced many chlorinated pesticides in the early to mid-20th century to slow their biodegradation in the field [19], but has now largely shifted over to PFCs [4]. The sheer number of PFCs produced, their widespread use and their apparent foreignness to microbes presents a large challenge for biodegradation. It is in this context that all current knowledge of metabolic activation and C–F bond cleavage chemistry must be applied to this problem.

## 2. Definitions of PFAS and PFCs

**Figure 1:**
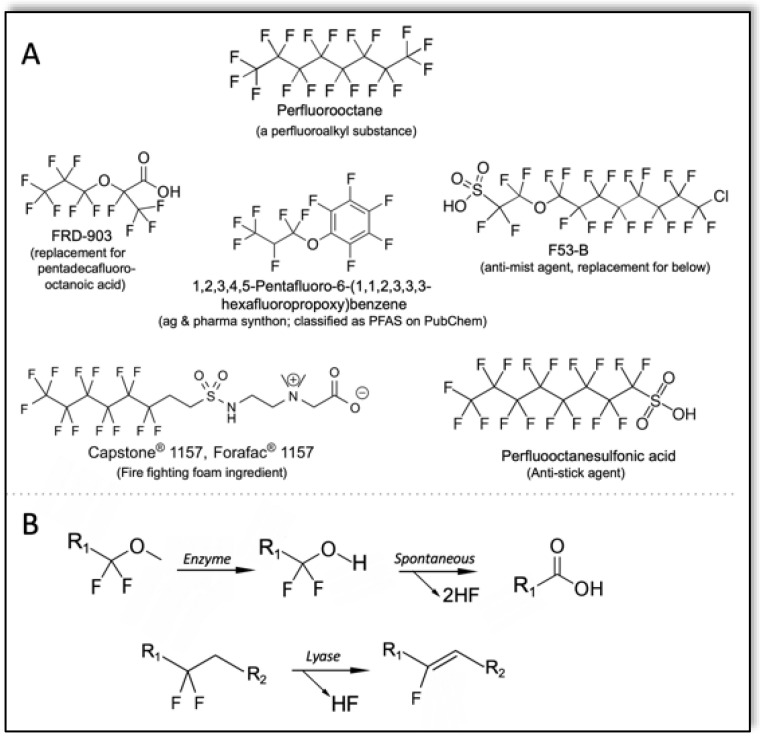
Polyflourinated organic compounds (PFCs) and mechanisms for their defluorination. (**A**) A perfluorinated alkane (top) and commercial PFCs showing the types of functionalities typically combined with perfluorinated moieties. (**B**) Two types of mechanisms by which a functionality adjacent to a perfluorinated carbon can be attacked enzymatically leading to facile defluorination.

The term PFAS (per- and polyfluoroalkyl substances) is used to refer to many different classes of PFCs with a wide divergence in their chemical reactivity. The ambiguity of the term has set up an unfortunate fuzziness in thinking about their biodegradation. For example, some compounds that meet the current Organization for Economic Co-operation and Development (OECD) definition of PFAS [20] are very biodegradable, while others are not. With few exceptions, the compounds referred to as **p**er- and **p**oly**f**luorinated **a**lkyl **s**ubstances (PFAS) contain aromatic rings, ether linkages and other functional groups that lead to a very large difference in biodegradability. An example of a perfluorinated alkane is octadecafluorooctane (perfluorooctane) (Figure 1A, top). Perfluoroalkanes have limited commercial uses compared to most compounds referred to as PFAS [21]. Virtually all of the regulated and targeted molecules for environmental remediation that are referred to as PFAS on PubChem [22] and other sites, contain functionalities in addition to their C–F bonds. Examples of some highly used and researched compounds are shown below the perfluoroalkane at the top of Figure 1A.

If the term PFAS has become too pervasive to avoid it, users should minimally state upfront which definition of PFAS they are using, as discussed in Barnabas, et al. [1]. OECD, the organization representing mostly European countries, has defined PFAS as any compound with at least a -CF_3_ group, or a -CF_2_- group that does not have a H/Cl/Br/I atom associated with it [20]. A new United States Environmental Protection Agency definition says that a PFAS is anything that contains (R_1_)(R_2_)(F)C–C(R_3_)F_2_ [1]. Barnabas and coworkers prefer the following PFAS definition: a compound that contains an (R_1_)(R_2_)(F)C–C(R_3_)F_2_ group where R groups are any atom except hydrogen and the bond between both aliphatic carbon atoms is a single bond. The Center for Disease Control (CDC) states, “PFAS are a group of chemicals used to make fluoropolymer coatings and products that resist heat, oil, stains, grease and water” [23]. Others have also questioned the definitions of PFAS for the difficulties that they pose for regulatory decisions [24]. Moreover, it has been pointed out that the currently marketed fluorinated pharmaceuticals are classified as 1% being PFAS by one definition, and as 100% being PFAS by another definition [25].

Depending on which definition one might choose, biodegradation of a designated PFAS chemical might be easy or hard, or difficult to pin down if you are talking about the biodegradation of chemicals that “resist heat.” In this review, the term “polyfluorinated organic compounds” is used, abbreviated as PFCs, and defines specific chemical classes in the context of the nuances of their biodegradation. The classes discussed are: perfluoroalkanes, substituted polyfluorinated alkanes, substituted polyfluorinated alkenes and substituted polyfluorinated aromatics.

## 3. Broad Principles for the Biodegradation of PFCs

Perfluorooctane presents a significant challenge for biodegradation relative to other PFCs (Figure 1). With perfluorooctane, chemical hydrolysis is not observed, and reduction of a C–F bond requires electrochemical potentials below −2000 mV [26]. More recent studies confirm these low potentials as being required [27,28]. The known lower limit for biological reduction potential attained by an enzymatic cofactor is −622 mV [29], making it unlikely that biology will evolve a new cofactor to poise its potential at −2000 mV. With additional functionality attached to a highly fluorinated, even perfluorinated, chain or ring, many other chemical and metabolic options are available to bring about defluorination. For example, the presence of an ether linkage with a C–O carbon containing a non-fluorine substituent presents a potential soft spot for enzyme cleavage that could produce a *gem*-difluoro alcohol that would undergo spontaneous elimination and hydrolysis to release fluoride. Moreover, such a metabolic step potentially sets up the molecule for further degradation proceeding down the alkyl or alkenyl chain, or into a ring (Figure 1B, top). An ether cleavage that leads to spontaneous defluorination would be plausible, for example, with FRD-903 and F53-B. Similarly, fluorotelomers like the firefighting foam shown in Figure 1A could plausibly be attacked by a lyase catalyzing elimination of fluoride (Figure 1B, bottom). The resultant olefin is susceptible to water addition, oxygenation or other reactions to continue metabolism. Indeed, microbial transformations of fluorotelomers have been described [30,31]. Bacteria carrying out these reactions include *Gordonia* sp. strain NB4-1Y, *Pseudomonas fluorescens* DSM 8341, *Pseudomonas oleovorans* and *Mycobacterium vaccae* JOB5.

Given that PFCs of the type shown in Figure 1 are very new to the biosphere, there has been little time on an evolutionary scale to put together consecutive reactions of the types shown to make dedicated metabolic pathways. This review discusses general principles as to how microbes may activate PFCs for defluorination by “attacking the weak points.” Since perfluorooctane and related perfluorinated alkanes are not the subject of any remediation activities this author is aware of, and virtually all molecules of interest have other functional groups, the dominant theme here is to attack non-fluorine functionalities and promote C–F bond cleavage indirectly. This review discusses the potential for this strategy in more detail, but first starts by describing the most well-understood example of direct enzymatic C–F bond cleavage.

## 4. Direct Enzymatic C–F Bond Cleavage Has Limitations

**Figure 2:**
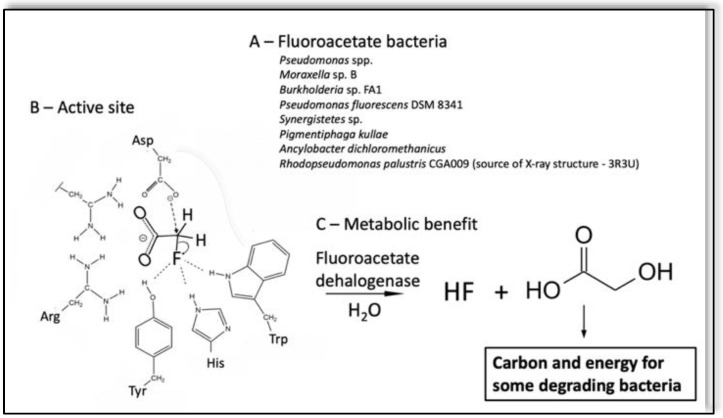
Fluoroacetate metabolism and enzyme from bacteria serves as the main, but rare, example of direct C–F bond cleavage. (**A**) Bacteria catalyzing defluorination of fluoroacetate. (**B**) Active site of the fluoroacetate dehalogenase from *Rhodospeudomonas palustris* CGA009 (3R3U). (**C**) Metabolic benefit. In addition to detoxification, some bacteria can use hydroxyacetic acid, or glycollate, as a sole source of carbon and energy to support growth.

Numerous bacteria have been isolated that defluorinate the widespread natural product fluoroacetate [32,33,34,35,36,37], largely mediated by fluoroacetate dehalogenase. This has become the mechanistic paradigm for direct S_N_2-type defluorination by a microbial enzyme (Figure 2) [38,39]. There are X-ray structures for fluoroacetate dehalogenases [39,40], and computational chemistry has been applied to better understand the power and limitations of the reaction [41,42,43]. The enzyme uses a carboxylate anion nucleophile. The negative charge on the substrate is shielded by the active site arginine and fluoride anion leaving is facilitated by a nearby triad of tyrosine, histidine and tryptophan residues. Water resolves the enzyme ester intermediately to produce the glycolic acid product. There is sufficient metabolic energy in glycolic acid to potentially support bacterial growth.

Perfluorinated acetic and longer chain carboxylic acids are not known to react readily, if at all, with fluoroacetate dehalogenase. Moreover, studies with wastewater microorganisms showed ready degradation of fluoroacetate, whereas di- and trifluoroacetate were recalcitrant [44]. Carbon–fluorine bond strength is known to increase with increasing fluorine substituents on an aliphatic carbon [45,46]. Moreover, unlike monofluoroacetate, di- and trifluoroacetate moieties are not known to be biosynthesized or found in natural products, hence microbial exposure to them has only occurred in recent history. Although fluoroacetate dehalogenase is a powerful enzyme, uniquely poised for direct defluorination [47], its limitations highlight the need to pursue different mechanistic strategies for biodegrading polyfluorinated compounds.

Given the observed hurdles in direct cleavage of unactivated C–F bonds, metabolic activation will likely be necessary to biodegrade many PFCs. This is not rare in biodegradative metabolism. As described in the next section, metabolic activation prior to degrading stable molecules is the norm rather than the exception in biodegradation.

## 5. Concept of Metabolic Activation in Biodegradation

The cost of acquiring additional genes, and hence new metabolic functions, is often given as a reason why microbial genome size has largely stayed within 1–10 Mb confines [48]. Each added gene requires more resources and time to replicate. Microbes sometimes acquire, and evolve, new genes via plasmids, transposons and mutations that create new functions [49,50,51]. This strategy may be evolutionarily efficient by keeping genomes streamlined, gaining valuable functions and shedding genes if they fail to provide sufficient survival benefit.

**Figure 3:**
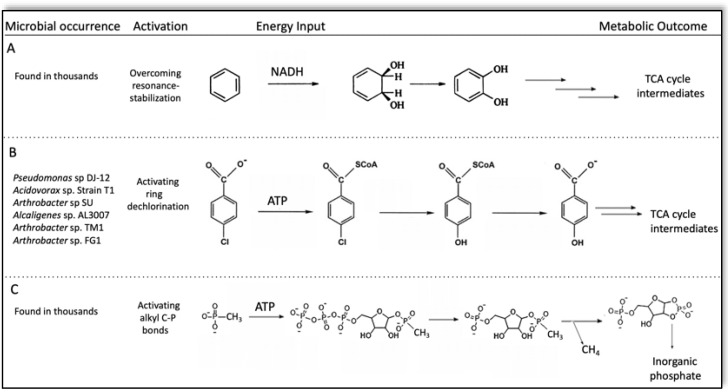
Examples of metabolic activation in microbial degradation pathways. (**A**) A major aerobic bacterial pathway for metabolizing benzenoid rings requires energy input to activate molecular oxygen for attack on the ring. The activated ring is then cleaved and fragments are assimilated in subsequent metabolism. (**B**) 4-Chlorobenzoate is activated for dichlorination via an initial energy-requiring thioesterification of the carboxylate moiety via coenzyme A. (**C**) The C–P bond of alkylphosphonates is relatively unreactive and is activated using ATP to accomplish C–P bond cleavage to release inorganic phosphate in phosphorus-poor environments.

Given the extreme selective pressures against the unrestrained expansion of metabolism, the common occurrence of metabolic activation attests to its importance. Metabolic activation refers to the initial steps in many biodegradative pathways in which energy or materials are not immediately gained. In many of those cases, the metabolic activation requires energy investment, which is later regained along with added energy and nutrients that support cell growth. In the present review, examples of metabolic activation are discussed that are most relevant to known and proposed microbial defluorination metabolism. Hydrocarbons and dialkyl ethers are common industrial materials, and so biodegradation of such chemicals has been studied extensively. In aerobic environments, aliphatic and aromatic hydrocarbons and ethers are often activated by oxygenases to produce alcohols, aldehydes and carboxylic acids that can enter intermediary metabolic pathways [52]. Enzymes that combine atmospheric oxygen with hydrocarbons must overcome a high energy barrier that requires activation of O_2_, typically with NAD(P)H (Figure 3A). Since there is often substantial energy gained from the complete aerobic oxidation of hydrocarbons and dialkyl ethers to carbon dioxide, the energy investment is more than compensated for.

An example of metabolic activation for dehalogenation has been observed with *p*-chlorobenzoate (Figure 3B) [53]. The carboxyl group is only mildly activating for the *para* position in nucleophilic aromatic substitution [54,55], causing some microorganisms to invest energy to make the *para* position more activated. This is achieved by transforming the carboxylate group into a coenzyme A-tethered thioether. The dechlorination can then proceed hydrolytically, and the ester is subsequently hydrolyzed to make the common growth substrate *p*-hydroxybenzoate. The thioesterification and its later hydrolytic cleavage costs a cell two ATP-equivalents per metabolic cycle due to the CoA ligase enzyme producing AMP and pyrophosphate. However, further of metabolism of *p*-hydroxybenzoate more than recoups the energy investment.

The cleavage of the C–P bond of alkyl phosphonates by C–P lyase provides an interesting parallel to plausible activation mechanisms for C–F bond cleavage (Figure 3C). The overall reaction stoichiometry for methyl phosphonate is: CH_3_-PO_3_^2−^ + H_2_O → CH_4_ + HOPO_3_^2−^. However, this is not a direct addition of water to the C–P bond; the bond is cleaved only following a set of activation reactions requiring ATP and six proteins, with the key steps shown in the figure [56,57]. The extensive biological investment in this reaction reflects the extreme chemical stability of alkylphosphonates, that are known to be stable to refluxing conditions in an acid or base [58]. However, methylphosphonate is now known to be highly abundant in marine environments where phosphate is scarce [59]. This has presumably driven the evolution of this complex biochemical machinery to biodegrade alkyl phosphonates for the release of the inorganic phosphate needed for cellular metabolism. The complexity of the physiology is underscored by the discovery that 14 gene products are encoded by the C–P lyase operon [60]. As shown in Figure 2C, the ultimate cleavage step requires ATP, is activated by a series of enzymatic steps and is ultimately driven by the stable formation of a cyclic phosphodiester with concomitant C–P bond breakage [56,57].

Other examples of metabolic activation to initiate biodegradation have been detailed in peer-reviewed literature [61,62]. Additionally, the present author perused the EAWAG-Biocatalysis/Biodegradation Database compilation of 219 biodegradation pathways [63] and found that ~2/3 showed an activation reaction(s) as initiating the pathway. Activation reactions are also apparent in the order of rules applied during biodegradation predictions [64] and in pesticide degradation records as captured on EnviPath [65]. Most compounds, for which biodegradation does not initiate with an activating reaction, are esters and amides that undergo simple hydrolysis reactions. For the majority of other metabolism requiring initial activations, oxygenase and CoA-ligase reactions are common. Other activating reactions include azo and nitro group reduction, phosphorylation of phenol during anaerobic biodegradation, aryl and aliphatic carboxylation and anaerobic ethylbenzene dehydrogenation.

The EAWAG-BBD lacks information on the biodegradation of PFCs by specific microbes and enzymes, and it does not predict defluorination reactions [66]. By contrast, the database contains information on more than twenty dechlorinating enzymes, some of which have available X-ray structures. Polychlorinated natural products are abundant in several environments, indicating millions of years of exposure to the biosphere, and some microorganisms have evolved to use them for their carbon and energy requirements [67]. This sometimes necessitates metabolic activation of the type highlighted in Figure 2B. Metabolic activation, by definition, requires multiply coordinated enzyme reactions that, in turn, requires multiple genes to be expressed simultaneously. This can be accomplished via operonic gene organization, such as for the 4-chlorobenzoate pathway (Figure 2B), or at least constitutive gene expression. The latter was observed for the atrazine-degradation plasmid in *Pseudomonas* sp. ADP in which a dechlorinase, and two subsequent enzymes in the pathway are encoded by non-regulated, dispersed genes flanked by transposons that show evidence of evolutionarily recent insertion into the plasmid [68]. Similar to the metabolism of recently introduced herbicides like atrazine, the biodegradation of PFCs is likely at an early stage of evolution. However, the development of biodegradative metabolism is rendered more difficult by the significantly greater strength of the C–F bond compared to the C–Cl bond, and so metabolic activation will more often be required to biodegrade PFCs.

## 6. Metabolic Activation of Trifluoromethyl Arenes for Indirect C–F Bond Cleavage

Evolution is at an immature stage with respect to the biodegradation of polyfluorinated compounds (PFCs), and in-depth mechanistic reports are lacking. This review draws on our limited knowledge of microbial PFC metabolism, and a more extensive knowledge of PFC chemistry. The first example given is for trifluoromethyl groups on benzenoid aromatic rings.

Trifluoromethyl arenes are common moieties in many agrichemicals and pharmaceuticals. One example, the antidepressant fluoxetine, more commonly known as Prozac, had already amassed more than $22 billion USD in sales by 2005 [69] and is increasingly detected in surface waters [70]. In 2010, 30% of blockbuster pharmaceuticals were fluorinated, most of them aromatic [71], and thirteen new fluorinated drugs were approved in 2020 alone by the United States Food and Drug Administration [72]. Despite the significance and increasing emergence of these chemicals, studies on their mechanisms of biodegradation are limited. In that context, this class serves as a major example of how biodegradation is most likely to proceed via metabolic activation to labilize the C–F bond, rather than carrying out a direct C–F bond cleavage on an unaltered pesticide or pharmaceutical.

**Figure 4:**
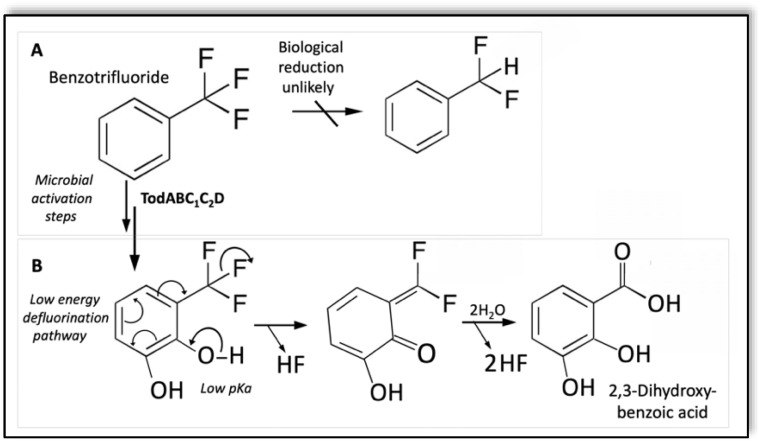
Metabolic activation of benzotrifluoride leading to complete defluorination of the trifluoromethyl group. (**A**) The direct cleavage of the C–F bond by a reductive mechanism is unlikely. (**B**) Dihydroxylation of the ring produces a phenolate anion at neutral pH that undergoes ring electron rearrangement that displaces one HF equivalent and produces an *ortho*-quinone methide. The latter may then undergo facile hydrolysis and complete defluorination.

It is instructive here to compare chemical and microbiological transformations of (trifluoromethyl)benzene, also known as benzotrifluoride (Figure 4). For example, the reduction potential for the direct C–F bond cleavage of benzotrifluoride, even with activating *para*-substituents, was below −1170 millivolts in all cases [73]. That potential is on the order of 500 millivolts below the lowest potential reported for a biological redox catalyst [29]. Yet, benzotrifluoride and their derivatives are reported to undergo defluorination by numerous microbes [74,75,76,77]. The mechanism is indirect, with metabolic activation occurring prior to C–F bond cleavage. The activation consists of the addition of oxygen to the ring that generates a low pK_a_ phenolic hydroxyl group *ortho* to the trifluoromethyl group, with which it acts synergistically (Figure 4). Facile spontaneous deprotonation of the *ortho*-phenolic group leads to double bond rearrangement to displace a fluoride anion and generate an *ortho*-quinone methide. The quinone methide is highly reactive to nucleophilic attack at the exocyclic double bond that can react with water in the aqueous environment of a bacterial cell to produce a *gem*-difluoro-benzyl alcohol. Further spontaneous defluorination in water produces a non-fluorinated benzoic acid.

Defluorination occurrence with microbially-produced trifluoromethyl catechols was first described in 1982 [74]. It has been described in cultures of *Rhodococcus rubropertinctus* N657 [75] and *Pseudomonas putida* F1 [77]. The defluorination in wild-type aromatic-degrading bacteria was dependent upon the inability of a catechol dioxygenase enzyme to process the trifluoromethyl catechol, but a higher yield could be accomplished in a recombinant *Escherichia coli* expressing toluene dioxygenase (TodABC_1_C_2_) and *cis*-dihydrodiol dehydrogenase (TodD) [77]. The defluorination is sufficiently facile to occur at neutral pH and 25 °C, suggestive that an evolved enzyme could catalyze highly efficient defluorination by facilitating *ortho*-hydroxyl deprotonation and adding water to the quinone methide.

## 7. Metabolic Activation of Chlorofluorocarbons for Indirect C–F Bond Cleavage

**Figure 5:**
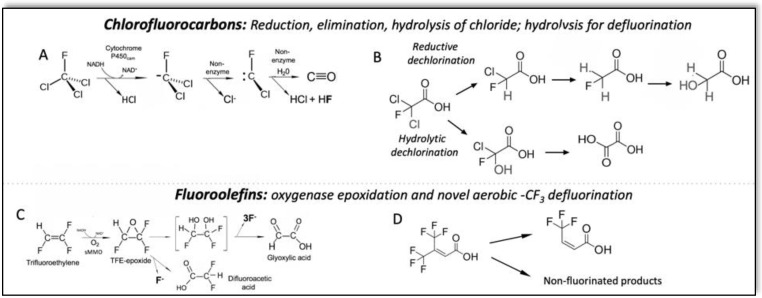
Metabolic activation in microbial defluorination. (**A**) Reductive dichlorination leading to ready defluorination catalyzed by the enzyme cytochrome P450_cam_ from *Pseudomonas putida* G7. (**B**) Reductive and hydrolytic declorination catalyzed by a microbial community leading to defluorination. (**C**) Fluoroolefin epoxidation leading to defluorination catalyzed by soluble methane monooxygenase from *Methylosinus trichosporium* OB3b. (**D**) Unusual displacement of a trifluoromethyl group from a polyfluorinated olefin by a microbial community, mechanism unknown.

The relative weakness of the C–Cl bond compared to the C–F bond provides a suitable weak entry point for metabolic activation, leading to defluorination. As an example, CF_4_ is highly inert but the refrigerant Freon-11, or trichlorofluoromethane was shown to undergo biotransformation via the heme enzyme cytochrome P450_cam_ from the bacterium *Pseudomonas putida* G7 [78]. Driven by NADH, the enzyme catalyzed an overall two-electron reduction to generate a dichlorofluoromethyl carbanion intermediate that rapidly and spontaneously eliminated a chloride anion (Figure 5A). The resultant fluorochlorocarbene is highly reactive with water and the expected defluorinated product, carbon monoxide, was demonstrated [78].

More recently, an anaerobic microbial-activated sludge community was tested with twelve chlorofluoro carboxylic acids from two to nine carbon atoms, and different ratios of chlorine to fluorine [79]. The reactivities leading to defluorination are illustrated with dichlorofluoroacetate (Figure 5B). Based on products detected, both reductive and hydrolytic dechlorination were indicated and led to defluorination of the C2 carboxylic acid, producing end products that could conceivably serve as growth substrates for bacteria.

## 8. Metabolic Activation of Fluoroolefins for Indirect C–F Bond Cleavage

Electron-withdrawing fluorine substituents on carbon–carbon double bonds render the double bond more susceptible to nucleophilic attack [80]. For example, perfluoroisobutene is so reactive with nucleophiles that it has been reported to react with solvents that are considered fairly inert, such as *N*,*N*-dimethylformamide [81]. Moreover, fluorine atoms, being smaller than chlorine or methyl group substituents, offer less steric hindrance to reactions with oxidizing agents, although reactions with electrophilic reagents are typically slowed. Tetrafluoroethylene is reported to react slowly but spontaneously with oxygen at low temperatures to give tetrafluoroethylene epoxide and uncharacterized polymeric materials [82]. Therefore, it is not surprising that an enzyme generating a highly reactive oxygen species [83] could react with a fluorinated ethylene. Trifluoroethylene was observed to react with the soluble methane monooxygenase from *Methylosinus trichosporium* OB3b, albeit at only 8% the rate observed with ethylene [84] (Figure 5C); novel reactions can be expected. A recent report described trifluoromethyl group elimination from a highly fluorinated olefin by an aerobic activated sludge community (Figure 5D) [85]. While single elimination from such trifluoromethylated olefins are well-known in the organic literature [86], the trifluoromethyl group elimination appears novel. Moreover, complete defluorination to small organic compounds was reported, a harbinger of further interesting microbial physiology.

## 9. Conclusions

In discussing biodegradability and mechanisms of biodegradation, it is preferable to use the term polyfluorinated compounds (PFCs), rather than PFAS, and to describe the structure of the molecule beyond the C–F bond. This review discusses strategies for the biodegradation of substituted polyfluorinated alkanes, alkenes and arenes and specific substituent impacts on C–F bond reactivity.

Considering biodegradation broadly, most environmental chemicals require initial metabolic activation prior to cleavage of the molecule into assimilatible fragments that provide benefit to the cell. Metabolic activation is particularly necessary as one considers plausible meachanisms for the biodegradation of PFCs. As the degree of fluorination increases, the direct reactivity of C–F bonds generally decreases. However, commercial PFCs almost invariably contain functionalities other than C–F bonds. Those accessory functional groups provide the potential for metabolic activation to render C–F bonds weaker and facilitate their cleavage. Typical activating reactions may set up a carbanion, a carbon radical, or facilitate electron flow to a fluorinated carbon by shifting ring currents. These strategies are most likely to prove fruitful for the biodegradation of PFCs in natural and engineered environments.

## Data Availability

Data discussed in the review are from, and available through, peer-reviewed published literature.

## References

[1] Barnabas S.J., Böhme T., Boyer S.K., Irmer M., Ruttkies C., Wetherbee I., Kondić T., Schymanski E.L., Weber L. (2022). Extraction of chemical structures from literature and patent documents using open access chemistry toolkits: A case study with PFAS. Digit. Discov..

[2] Evich M.G., Davis M.J.B., McCord J.P., Acrey B., Awkerman J.A., Knappe D.R.U., Lindstrom A.B., Speth T.F., Tebes-Stevens C., Strynar M.J. (2022). Per- and polyfluoroalkyl substances in the environment. Science.

[3] Ogawa Y., Tokunaga E., Kobayashi O., Hirai K., Shibata N. (2020). Current contributions of organofluorine compounds to the agrochemical industry. Iscience.

[4] Alexandrino D.A., Almeida C.M.R., Mucha A.P., Carvalho M.F. (2022). Revisiting pesticide pollution: The case of fluorinated pesticides. Environ. Pollut..

[5] Inoue M., Sumii Y., Shibata N. (2020). Contribution of organofluorine compounds to pharmaceuticals. ACS Omega.

[6] Liu J., Avendaño S.M. (2013). Microbial degradation of polyfluoroalkyl chemicals in the environment: A review. Environ. Int..

[7] Ochoa-Herrera V., Field J.A., Luna-Valesco A., Sierra-Alvarez R. (2016). Microbial toxicity and biodegradability of perfluorooctane sulfonate (PFOS) and shorter chain perfluoroalkyl and perfluoroalkyl substances (PFASs). Environ. Sci. Process Impacts.

[8] Zhang W., Pang S., Lin Z., Mishra S., Bhatt P., Chen S. (2021). Biotransformation of perfluoroalkyl acid precursors from various environmental systems: Advances and perspectives. Environ. Pollut..

[9] Zhang Z., Sarkar D., Biswas J.K., Datta R. (2022). Biodegradation of per- and polyfluoroalkyl substances (PFAS): A review. Bioresour Technol..

[10] Weber E.J., Tebes-Steves C., Washington J.W., Gladstone R. (2022). Development of a PFAS reaction library: Identifying plausible transformation pathways in environmental and biological systems. Environ. Sci. Process Impacts.

[11] Carvalho M.F., Alves C.C., Ferreira M.I., De Marco P., Castro P.M. (2022). Isolation and initial characterization of a bacterial consortium able to mineralize fluorobenzene. Appl. Environ. Microbiol..

[12] Finkelstein Z.I., Baskunov B.P., Boerrsma M.G., Vervoot J., Golovlev E.L., van Berkel W.J., Govleva L.A., Riietjens I.M. (2000). Identification of fluoropyrogallols as new intermediates in biotransformation of monofluorophenols in *Rhodococcus opacus* 1cp. Appl. Environ. Microbiol..

[13] Vargas C., Song B., Camps M., Häggblom M.M. (2000). Anaerobic degradation of fluorinated aromatic compounds. Appl. Microbiol. Biotechnol..

[14] Turner A.M., Abplanalp M.J., Bergantini A., Frigge R., Zhu C., Sun B.J., Hsiao C.T., Chang A.H., Meinert C., Kaiser R.I. (2019). Origin of alkylphosphonic acids in the interstellar medium. Sci. Adv..

[15] Wackett L.P., Robinson S.L. (2020). The ever-expanding limits of enzyme catalysis and biodegradation: Polyaromatic, polychlorinated, polyfluorinated, and polymeric compounds. Biochem. J..

[16] Harper D.B., O’Hagan D., Murphy C.D. (2003). Fluorinated natural products: Occurrence and biosynthesis. Nat. Prod. Organohalogen Compd..

[17] Walker M.C., Chang M.C. (2014). Natural and engineered biosynthesis of fluorinated natural products. Chem. Soc. Rev..

[18] Gribble G.W. (2015). A recent survey of naturally occurring organohalogen compounds. Environ. Chem..

[19] Rosner D., Markowitz G. (2013). Persistent pollutants: A brief history of the discovery of the widespread toxicity of chlorinated hydrocarbons. Environ. Res..

[20] Wang Z., Buser A.M., Cousins I.T., Demattio S., Drost W., Johansson O., Ohno K., Patlewicz G., Richard A.M., Walker G.W. (2021). A new OECD definition for per-and polyfluoroalkyl substances. Environ. Sci. Technol..

[21] Freire M.G., Gomes L., Santos L.M., Marrucho I.M., Coutinho J.A. (2006). Water solubility in linear fluoroalkanes used in blood substitute formulations. J. Phys. Chem. B.

[22] Kim S., Chen J., Cheng T., Gindulyte A., He J., He S., Li Q., Shoemaker B.A., Thiessem P.A., Yu B. (2021). PubChem in 2021: New data content and improved web interfaces. Nucleic Acids Res..

[23] CDC, Per- and Polyfluorinated Substances (PFAS) Factsheet. https://www.cdc.gov/biomonitoring/PFAS_FactSheet.html.

[24] Wallington T.J., Andersen M.S., Nielsen O.J. (2021). The case for a more precise definition of regulated PFAS. Env. Sci. Proc. Impacts.

[25] Hammel E., Webster T.F., Gurney R., Heiger-Bernays W. (2022). Implications of PFAS definitions using fluorinated pharmaceuticals. Iscience.

[26] Pud A.A., Shapoval G.S., Kukhar V.P., Mikulina O.E., Gervits L.L. (1995). Electrochemical reduction of some saturated and unsaturated perfluorocarbons. Electrochim. Acta.

[27] Combellas C., Kanoufi F., Thiebault A. (2003). Reduction of polyfluorinated compounds. J. Phys. Chem. B.

[28] Houmam A. (2008). Electron transfer initiated reactions: Bond formation and bond dissociation. Chem. Rev..

[29] Huwiler S.G., Löffler C., Anselmann S.E., Stärk H.J., von Bergen M., Flechsler J., Rachel R., Boll M. (2019). One-megadalton metalloenzyme complex in *Geobacter metallireducens* involved in benzene ring reduction beyond the biological redox window. Proc. Natl. Acad. Sci. USA.

[30] Kim M.H., Wang N., Chu K.H. (2014). 6:2 Fluorotelomer alcohol (6:2 FTOH) biodegradation by multiple microbial species under different physiological conditions. Appl. Microbiol. Biotechnol..

[31] Shaw D.M., Munoz G., Bottos E.M., Duy S.V., Sauvé S., Liu J., Van Hamme J.D. (2019). Degradation and defluorination of 6: 2 fluorotelomer sulfonamidoalkyl betaine and 6: 2 fluorotelomer sulfonate by *Gordonia* sp. strain NB4-1Y under sulfur-limiting conditions. Sci.Total Environ..

[32] Goldman P. (1965). The enzymatic cleavage of the carbon-fluorine bond in fluoroacetate. J. Biol. Chem..

[33] Liu J.Q., Kurihara T., Ichiyama S., Miyagi M., Tsunasawa S., Kawasaki H., Soda K., Esaki N. (1998). Reaction mechanism of fluoroacetate dehalogenase from *Moraxella* sp. B. J. Biol. Chem..

[34] Kurihara T., Yamauchi T., Ichiyama S., Takahata H., Esaki N. (2003). Purification, characterization, and gene cloning of a novel fluoroacetate dehalogenase from *Burkholderia* sp. FA1. J. Mol. Catal. B Enzym..

[35] Donnelly C., Murphy C.D. (2009). Purification and properties of fluoroacetate dehalogenase from *Pseudomonas fluorescens* DSM 8341. Biotechnol. Lett..

[36] Davis C.K., Webb R.I., Sly L.I., Denman S.E., McSweeney C.S. (2012). Isolation and survey of novel fluoroacetate-degrading bacteria belonging to the phylum Synergistetes. FEMS Microbiol. Ecol..

[37] Camboim E.K., Almeida A.P., Tadra-Sfeir M.Z., Junior F.G., Andrade P.P., McSweeney C.S., Melo M.A., Riet-Correa F. (2012). Isolation and identification of sodium fluoroacetate degrading bacteria from caprine rumen in Brazil. Sci. World J..

[38] Chan W.Y., Wong M., Guthrie J., Savchenko A.V., Yakunin A.F., Pai E.F., Edwards E.A. (2010). Sequence-and activity-based screening of microbial genomes for novel dehalogenases. Microb. Biotechnol..

[39] Chan P.W., Yakunin A.F., Edwards E.A., Pai E.F. (2011). Mapping the reaction coordinates of enzymatic defluorination. J. Am. Chem. Soc..

[40] Jitsumori K., Omi R., Kurihara T., Kurata A., Mihara H., Miyahara I., Hirotsu K., Esaki N. (2009). X-Ray crystallographic and mutational studies of fluoroacetate dehalogenase from *Burkholderia* sp. strain FA1. J. Bacteriol..

[41] Kamachi T., Nakayama T., Shitamichi O., Jitsumori K., Kurihara T., Esaki N., Yoshizawa K. (2009). The catalytic mechanism of fluoroacetate dehalogenase: A computational exploration of biological dehalogenation. Chemistry.

[42] Miranda-Rojas S., Fernández I., Kästner J., Toro-Labbé A., Mendizabal F. (2018). Unraveling the nature of the catalytic power of fluoroacetate dehalogenase. ChemCatChem.

[43] Kang H., Zheng M. (2021). Influence of the quantum mechanical region size in QM/MM modelling: A case study of fluoroacetate dehalogenase catalyzed CF bond cleavage. Comp. Theor. Chem..

[44] Alexandrino D.A.M., Ribeiro I., Pinto L.M., Cambra R., Oliveira R.S., Pereira F., Carvalho M.F. (2018). Biodegradation of mono-, di- and trifluoroacetate by microbial cultures with different origins. New Biotechnol..

[45] Dolbier W.R. (2005). Fluorine chemistry at the millenium. J. Fluor. Chem..

[46] O’Hagan D. (2008). Understanding organofluorine chemistry. An introduction to the C-F bond. Chem. Soc. Rev..

[47] Chan P.W., Chakrabarti N., Ing C., Halgas O., To T.K., Wälti M., Petit A.P., Tran C., Savchenko A., Yakunin A.F. (2022). Defluorination capability of l-2-haloacid dehalogenases in the HAD-like hydrolase superfamily correlates with active site compactness. ChemBioChem.

[48] Sela I., Wolf Y.I., Koonin E.V. (2016). Theory of prokaryotic genome evolution. Proc. Natl. Acad. Sci. USA.

[49] Wackett L.P. (2004). Evolution of enzymes for the metabolism of new chemical inputs into the environment. J. Biol. Chem..

[50] Davidi D., Longo L.M., Jabłonńska J., Milo R., Tawfik D.S. (2018). A bird’s-eye view of enzyme evolution: Chemical, physicochemical, and physiological considerations. Chem. Rev..

[51] Copley S.D. (2021). Setting the stage for evolution of a new enzyme. Curr. Opin. Struct. Biol..

[52] Gibson D.T., Parales R.E. (2000). Aromatic hydrocarbon dioxygenases in environmental biotechnology. Curr. Opin. Biotechnol..

[53] Dunaway-Mariano D., Babbitt P.C. (1994). On the origins and functions of the enzymes of the 4-chlorobenzoate to 4- hydroxybenzoate converting pathway. Biodegradation.

[54] Bunnett J.F., Morath R.J., Okamoto T. (1955). The ortho:para ratio in activation of aromatic nucleophilic substitution by the carboxylate group. J. Am. Chem. Soc..

[55] Terrier F. (2013). Modern Nucleophilic Aromatic Substitution.

[56] Kamat S.S., Raushel F.M. (2013). The enzymatic conversion of phosphonates to phosphate by bacteria. Curr. Opin. Chem. Biol..

[57] Yang K., Ren Z., Raushel F.M., Zhang J. (2016). Structures of the carbon-phosphorus lyase complex reveal the binding mode of the nbd-like phnk. Structure.

[58] Hoskin F.C.G. (1955). Some observations concenring the biochemical inertness of methylphosphonic and isopropyl methylphosphonic acids. Can. J. Biochem. Physiol..

[59] Metcalf W.W., Griffin B.M., Cicchillo R.M., Gao J., Janga S.C., Cooke H.A., Circello B.T., Evans B.S., Martens-Habbena W., Stahl D.A. (2012). Synthesis of methylphosphonic acid by marine microbes: A source for methane in the aerobic ocean. Science.

[60] Chen C.M., Ye Q.Z., Zhu Z.M., Wanner B.L., Walsh C.T. (1990). Molecular biology of carbon-phosphorus bond cleavage. Cloning and sequencing of the *phn* (*psiD*) genes involved in alkylphosphonate uptake and C-P lyase activity in *Escherichia coli* B. J. Biol. Chem..

[61] VanBriesen J.M. (2001). Thermodynamic yield predictions for biodegradation through oxygenase activation reactions. Biodegradation.

[62] Coates J.D., Chakraborty R., McInerney M.J. (2002). Anaerobic benzene biodegradation—A new era. Res. Microbiol..

[63] Gao J., Ellis L.B., Wackett L.P. (2010). The University of Minnesota biocatalysis/biodegradation database: Improving public access. Nucleic Acids Res..

[64] Fenner K., Gao J., Kramer S., Ellis L., Wackett L.P. (2008). Data-driven extraction of relative reasoning rules to limit combinatorial explosion in biodegradation pathway prediction. Bioinformatics.

[65] Latino D.A., Wicker J., Gütlein M., Schmid E., Kramer S., Fenner K. (2017). Eawag-Soil in enviPath: A new resource for exploring regulatory pesticide soil biodegradation pathways and half-life data. Environ. Sci. Proc. Impacts.

[66] Gao J., Ellis L.B., Wackett L.P. (2011). The University of Minnesota Pathway Prediction System: Multi-level prediction and visualization. Nucleic Acids Res..

[67] Holliger C., Wohlfarth G., Diekert G. (1998). Reductive dechlorination in the energy metabolism of anaerobic bacteria. FEMS Microbiol. Rev..

[68] Martinez B., Tomkins J., Wackett L.P., Wing R., Sadowsky M.J. (2001). Complete nucleotide sequence and organization of the atrazine catabolic plasmid pADP-1 from *Pseudomonas* sp. strain ADP. J. Bacteriol..

[69] Wenthur C.J., Bennett M.R., Lindsley C.W. (2013). Classics in Chemical Neuroscience: Fluoxetine (Prozac). ACS Chem. Neurosci..

[70] Martin J.M., Bertram M.G., Saaristo M., Fursdon J.B., Hannington S.L., Brooks B.W., Burket S.R., Mole R.A., Deal N.D., Wong B.B. (2019). 2019. Antidepressants in surface waters: Fluoxetine influences mosquitofish anxiety-related behavior at environmentally relevant levels. Environ. Sci. Technol..

[71] O’Hagan D. (2010). Fluorine in health care: Organofluorine containing blockbuster drugs. J. Fluor. Chem..

[72] Yu Y., Liu A., Dhawan G., Mei H., Zhang W., Izawa K., Soloshonok V.A., Han J. (2021). Fluorine-containing pharmaceuticals approved by the FDA in 2020: Synthesis and biological activity. Chin. Chem. Lett..

[73] Combellas C., Kanoufi F., Thiébault A. (1996). Reducibility of the carbon-fluorine bond in the trifluoromethyl group. J. Electroanal. Chem..

[74] Engesser K.H. (1982). Der Einfluss der Trifluormethylgruppe auf die Biologische Abbaubarkeit von Aromaten. Doctoral Dissertation.

[75] Engesser K.H., Cain R.B., Knackmuss H.J. (1988). Bacterial metabolism of side chain fluorinated aromatics: Cometabolism of 3- trifluoromethyl(TFM)-benzoate by *Pseudomonas putida* (*arvilla*) mt-2 and *Rhodococcus rubropertinctus* N657. Arch. Microbiol..

[76] Kiel M., Engesser K.H. (2015). The biodegradation vs. biotransformation of fluorosubstituted aromatics. Appl. Microbiol. Biotechnol..

[77] Bygd M.D., Aukema K.G., Richman J.E., Wackett L.P. (2022). Microwell fluoride screen for chemical, enzymatic, and cellular reactions reveals latent microbial defluorination capacity for -CF3 groups. Appl. Environ. Microbiol..

[78] Li S., Wackett L.P. (1993). Reductive dehalogenation by cytochrome P450CAM: Substrate binding and catalysis. Biochemistry.

[79] Jin B., Che S., Gao J., Yu Y., Liu J., Men Y. (2022). Anaerobic defluorination of chlorine- substituted per- and polyfluorinated carboxylic acids triggered by microbial dechlorination. ChemRxiv.

[80] Timperley C.M., Waters M.J., Greenall J.A. (2006). Fluoroalkene chemistry: Part 3. Reactions of arylthiols with perfluoroisobutene, perfluoropropene and chlorotrifluoroethene. J. Fluor. Chem..

[81] Zeifman Y.V., Ter-Gabrielyan E.G., Gambaryan N.P., Knunyants I.L. (1984). The chemistry of perfluoroisobutene. Russ. Chem Rev..

[82] Gozzo F., Camaggi G. (1996). Oxidation reactions of tetrafluoroethylene and their products—I: Auto-oxidation. Tetrahedron.

[83] Wallar B.J., Lipscomb J.D. (1996). Dioxygen activation by enzymes containing binuclear non-heme iron clusters. Chem. Rev..

[84] Fox B., Borneman J.G., Wackett L.P., Lipscomb J.D. (1990). Haloalkene oxidation by the soluble methane monooxygenase from *Methylosinus trichosporium* OB3b: Mechanistic and environmental implications. Biochemistry.

[85] Yu Y., Che S., Ren C., Bosen J., Zhenyu T., Liu J., Men Y. (2022). Microbial defluorination of unsaturated per- and polyfluorinated carboxylic acids under anaerobic and aerobic conditions: A structure specificity study. Environ. Sci. Technol..

[86] Liu Y., Zhou Y., Zhao Y., Qu J. (2017). Synthesis of gem-difluoroallylboronates via FeCl2-catalyzed boration/β-fluorine elimination of trifluoromethyl alkenes. Org. Lett..

